# Changes in morphological and physiological traits of urban trees in response to elevated temperatures within an Urban Heat Island

**DOI:** 10.1093/treephys/tpae145

**Published:** 2024-11-14

**Authors:** Johanna Andrea Martínez-Villa, Alain Paquette, Kenneth J Feeley, Paula Andrea Morales-Morales, Christian Messier, Sandra M Durán

**Affiliations:** Département des sciences biologiques, Centre for Forest Research, Université du Québec à Montréal, Montréal, Québec, 141 Av. du Président-Kennedy, Montréal, QC H2X 1Y4, Canada; Département des sciences biologiques, Centre for Forest Research, Université du Québec à Montréal, Montréal, Québec, 141 Av. du Président-Kennedy, Montréal, QC H2X 1Y4, Canada; Biology Department, University of Miami, 1301 Memorial Dr #215, Coral Gables, FL 33146, United States; Departamento de Ciencias Forestales, Universidad Nacional de Colombia – Sede Medellín, Medellin, Antioquia, Cra. 65 #59a-110, Medellín, Antioquia, Colombia; Département des sciences biologiques, Centre for Forest Research, Université du Québec à Montréal, Montréal, Québec, 141 Av. du Président-Kennedy, Montréal, QC H2X 1Y4, Canada; Department of Forest and Rangeland Stewardship, Colorado State University, 1472 Campus DeliveryFort Collins, CO 80523-1472, United States

**Keywords:** climate change, functional traits, photosynthesis, physiological responses, thermal tolerance, urban forest

## Abstract

Urban heat islands (UHIs) are a common phenomenon in metropolitan areas worldwide where the air temperature is significantly higher in urban areas than in surrounding suburban, rural or natural areas. Mitigation strategies to counteract UHI effects include increasing tree cover and green spaces to reduce heat. The successful application of these approaches necessitates a deep understanding of the thermal tolerances in urban trees and their susceptibility to elevated urban temperatures. We evaluated how the photosynthetic thermal optimum (T_opt_), photosynthetic heat tolerance (T_50_) and key leaf thermoregulatory morphological traits (leaf area [LA], specific leaf area, leaf width, thickness and leaf dry matter content) differ between conspecific trees growing in ‘hot’ (UHI) vs ‘cool’ parts of Montreal, Canada (with a difference of 3.4 °C in air temperature), to assess the ability of seven common tree species to acclimation to higher temperatures. We hypothesized that individuals with hotter growing temperatures would exhibit higher T_opt_ and T_50_, as well as leaf thermoregulatory morphological traits aligned with conservative strategies (e.g., reduced LA and increased leaf mass) compared with their counterparts in the cooler parts of the city. Contrary to our a priori hypotheses, LA increased with growing temperatures and only four of the seven species had higher T_50_ and only three had higher T_opt_ values in the hotter area. These results suggest that many tree species cannot acclimate to elevated temperatures and that the important services they provide, such as carbon capture, can be negatively affected by high temperatures caused by climate change and/or the UHI effect. The ability vs inability of tree species to acclimate to high temperatures should be considered when implementing long term tree planting programs in urban areas.

## Introduction

Global warming and urbanization expose organisms to unique environmental conditions (IPCC 2022). Urban areas have high surface and air temperatures, with temperatures generally increasing from rural areas to more densely urbanized city cores. This urban heat island (UHI) effect exacerbates heat stress, risking urban infrastructure and human populations (Tuholske et al. 2021). Mitigation strategies such as the creation of parks and increasing tree cover have been proposed to counteract the UHI effect (Karimi et al. 2022). Nonetheless, the efficacy of these strategies may be attenuated by the physiological tolerances of trees and their ability to avoid heat stress (Teskey et al. 2015). To avoid heat stress, urban trees must acclimate to higher temperatures through changes in their physiology (e.g., photosynthesis thermal optima or photosynthetic heat tolerance) (Hara et al. 2021) and/or by adjusting leaf traits to reduce leaf surface temperatures (Zhu et al. 2020). Unfortunately, there is limited information on how trees respond to urban temperatures (Kullberg and Feeley 2022), limiting our ability to predict their vulnerability to UHIs in a warming world (Esperon-Rodriguez et al 2022).

Rather than air temperature, leaf temperature is critical for leaf physiological processes (Cavaleri 2020). Plants may exhibit plastic responses in leaves to acclimate to warmer environments, maintain leaf temperature below damage thresholds and optimize net photosynthetic CO_2_ uptake (A_net_) (Way and Yamori 2014, Crous et al. 2022). Leaf thermoregulation is mediated, in part, by the morphological leaf traits affecting heat dissipation (Leigh et al. 2017, Fauset et al. 2018). Traits such as leaf area (LA), effective leaf width (LW), leaf thickness (LT), specific leaf area (SLA) and leaf dry matter content (LDMC) determine leaf temperature and therefore its thermal dynamics (Michaletz et al. 2015). In other words, species with different leaf traits growing under the same air temperature may experience different leaf temperatures. For instance, large, thin leaves typically reach higher temperatures than smaller, thicker leaves due to their thicker boundary layer (which influences the heat transfer) and their low water content (which increases the rate of leaf temperature change) (Leigh et al. 2017, Tserej and Feeley 2021). Nonetheless, trees can adjust their morphological traits to different air temperatures, as evidenced studies of decreased leaf size with increasing temperatures (Zhu et al. 2020, Manishimwe et al. 2022). These adjustments enable passive thermoregulation, maintaining homeothermy by influencing leaf energy balance and heat dissipation (Michaletz et al. 2015).

Effective acclimation to higher temperatures should lead to a low or even negative difference between leaf and air temperature (ΔT), where leaves remain cooler than the air due to high stomatal conductance to water vapor (g_s_) and transpiration (E) at high temperatures (Blonder et al. 2020). The energy balance theory, useful for modeling leaf temperatures, incorporates environmental factors such as solar radiation, wind, relative humidity, and morphological and physiological leaf traits such as g_s_, E and LW (Michaletz et al. 2015), enabling the understanding of how plants adjust and compensate their traits to regulate internal leaf temperature depending on the environment (Vårhammar et al. 2015). For instance, studies have shown that reducing leaf size and increasing E improve ΔT (Vårhammar et al. 2015, Tarvainen et al. 2022). Investigating the ΔT between individuals growing in different urban environments, especially within UHIs, offers valuable insights into the ability of a species to avoid extreme leaf temperatures.

Photosynthesis is especially sensitive to temperature variation, peaking at a species-specific optimum before declining at higher temperatures (Vårhammar et al. 2015, Tarvainen et al. 2022). The optimal temperature for photosynthesis (T_opt_) aligns with the local daytime temperatures, suggesting acclimation or local adaptation (Vårhammar et al. 2015). In urban environments, we might expect trees within the hotter areas to have both higher T_opt_ and A_net_ (constructive photosynthetic adjustments) if they acclimate effectively to elevated growing temperatures caused by the UHI effect. Similarly, photosystem II (PSII) is highly heat sensitive (Baker 2008). Photosynthetic heat tolerances (P_HT_), defined as the temperatures causing irrecoverable PSII damage, can be characterized by the leaf critical temperature (T_crit_) and the leaf thermal tolerance (T_50_ and T_95_), which indicate the temperatures at which PSII have initial, 50 or 95% of damage, respectively, are commonly used to predict vulnerability to heat damage (Perez and Feeley 2020). Thus, higher values of T_crit_, T_50_ and T_95_ in the UHI show the P_HT_ acclimation in response to elevated temperatures. Although prior studies in urban settings have demonstrated T_opt_ and P_HT_ acclimation to high temperatures, much of the research has focused on controlled conditions in small trees (Hara et al. 2021) and less is understood about how trees respond to increases in temperature typical of UHI effects. There is therefore a pressing need for further investigation into the long-term acclimation of photosynthetic heat tolerances in mature trees under actual urban conditions.

Here, we evaluated the acclimation capacity of seven urban tree species to higher contrasting environments within a temperate city, representing the hottest (UHI) and coldest temperature extremes. We addressed the following questions: (QI) is there a response of urban trees to the elevated temperatures within the UHI in their morphological or physiological traits? We hypothesized a priori that, (HI) If urban trees exhibit distinct morphological and physiological acclimation in response to elevated temperatures within UHIs, traits should exhibit significative differences between the UHI and cooler urban environments.

(QII) Are changes in leaf morphological traits allowing for passive thermoregulation within UHIs. (HII) At higher temperatures, we expect smaller, narrower and thicker leaves (lower SLA, LA and LW) with high dry matter content (LDMC) and thickness (LT). These changes will enhance cooling due to a smaller boundary layer allowing for increased vapor diffusion. On the other hand, larger LA and LW can be expected when plants are increasing the surface area to increase E and g_s_.

(QIII) Is the ΔT be lower for individuals grown under higher temperatures? (HIII) If leaves acclimate their leaf traits as predicted under HI and HII, we expect a smaller difference between leaf and air temperature for trees growing in the UHI vs cooler parts of the city.

(QIV) Do trees acclimate to higher temperatures within UHIs through physiological changes in their photosynthetic thermal optimum (T_opt_) and/or photosynthetic thermal tolerances (P_HT_)? (HIV) Since individuals have developed all their life under within the UHI, they will have higher T_opt_ and/or P_TH_ than individuals from cooler parts of the city.

## Materials and methods

### Study area

We conducted the project in the city of Montreal, Canada from July to September 2021. The city has a warm-summer and humid continental climate (Beck et al. 2018). During the study period, precipitation ranged from 173.1 to 364.6 mm, with July being the wettest month (https://climate.weather.gc.ca/). The annual precipitation in 2021 was 811.7 mm; 200 mm less than the multiyear average (https://montreal.weatherstats.ca/charts/precipitation-yearly.html). Furthermore, temperatures record of 37.8 ± 2.3 °C in the UHI and 34.4 ± 2.1 °C in the coldest part, thus, maximum temperatures showed a warming of 3.4 °C in the UHI. Mean temperatures were ~23.3 ± 4.3 °C and ~22.0 ± 4.6 °C for the UHI and the coldest part of the city, with a warming of 1.3 °C (Table S1 available as Supplementary data at *Tree Physiology* Online). The highest temperatures were recorded in August.

**Figure 1:**
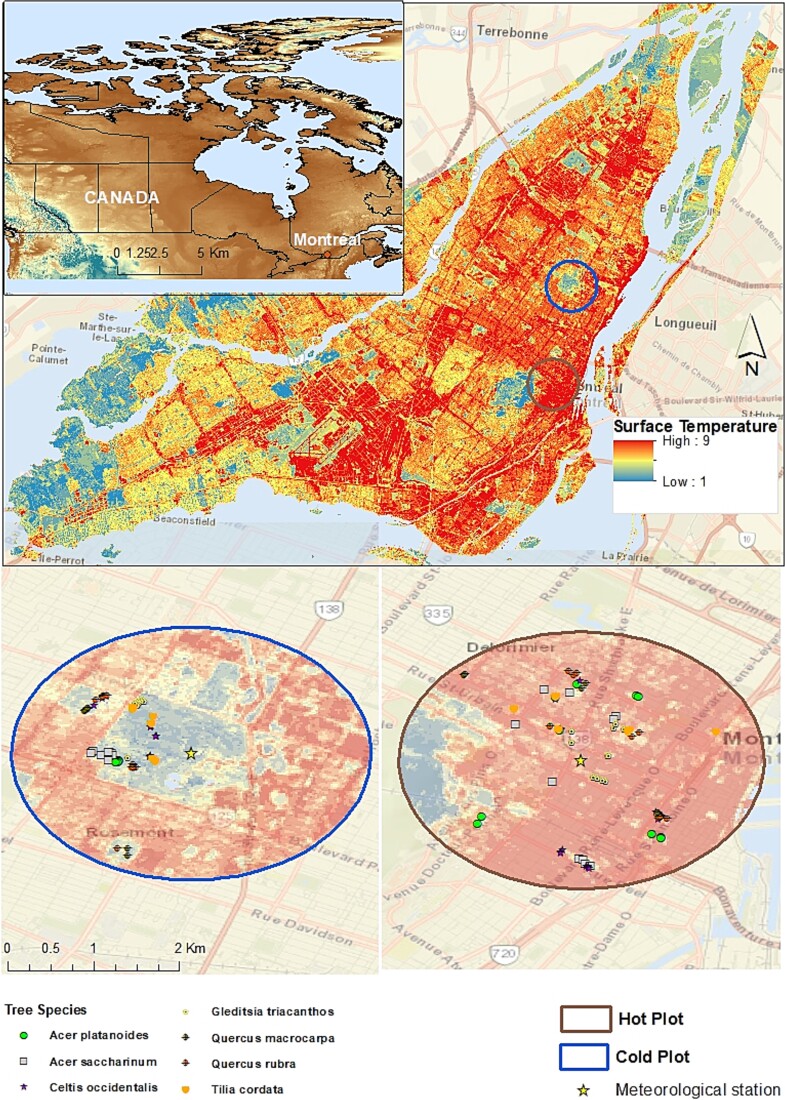
Location of the two urban environments within the city of Montreal. The hottest part of the city (UHI) is located in the downtown (Hot plot) and the coolest part is located in the Botanical Garden (Cold plot). Tree species are represented by symbols and stars representing the meteorological station from the municipality in each environment.

To identify study sites in the city with the greatest temperature disparities, we referred to Montreal’s surface temperature map based on satellite images of 2020 (https://open.canada.ca/data/en/dataset/dbdfbdba-0725-470d-a23e-da69dbedc4e6). We selected two distinct environments for study: the downtown area as the hottest part (the UHI) and the Botanical Garden of Montreal as the coldest part in the city (Figure 1). The differences between the two urban environments are 1.3 and 3.4 °C in mean and maximum air temperature, respectively. The geographical distance between our two urban environments is ~ 6.5 km to maintain similar relative humidity and precipitation conditions and focus on temperature contrast. The UHI effect in Montreal has been extensively studied, primarily using ground surface temperature data (Boulfroy et al. 2012, Touchaei and Wang 2015, Wang and Akbari 2016, Roberge and Sushama 2018). As a result, the hottest areas in the city are well-identified. To capture detailed micro-climatic conditions within each urban environment, we installed five HOBO data loggers (Onset UA-002-64) during field data collection, recording air temperature and solar radiation every 30 min.

We deliberately chose public parks in the downtown area and the Botanical Garden due to the absence of management actions (watering or fertilization), enhancing the similarity between these two sites. To further enhance the comparability of habitat conditions between environments, we specifically focused on park trees and excluded street trees in the downtown area.

### Thermoregulatory morphological leaf traits

We selected the seven most common tree species across our two urban environments: *Acer platanoides* (ACPL)*, Acer saccharinum* (ACSA)*, Celtis occidentalis* (CEOC)*, Gleditsia triacanthos* (GLTR)*, Quercus macrocarpa* (QUMA)*, Quercus rubra* (QURU) and *Tilia cordata* (TICO)*.*

We measured six traits important for leaf thermoregulation following the protocol from Pérez-Harguindeguy et al. (2016): LA, effective LW, LT, SLA, LDMC and leaf absorptance to shortwave radiation.

Leaf area and effective LW are crucial traits in determining leaf temperature. Leaf area is a robust predictor of temperature range and variation within the leaf (Leigh et al. 2017, Tserej and Feeley 2021), while LW strongly influences the leaf boundary layer size and the photosynthetic surface (Leigh et al. 2017). A larger LW, for instance, leads to increased boundary layer resistance, limiting the exchange of heat and water vapor between the leaf and the environment. Additionally, wider and larger leaves have a higher capacity to intercept and absorb solar radiation, resulting in an elevated leaf temperature. Nevertheless, wider leaves generally exhibit a larger surface available for transpiration, promoting cooling in species with high transpiration rates (Fauset et al. 2018).

Leaf thickness is a critical trait for thermal tolerance since it determines the leaf’s thermal mass, which plays a vital role in the species’ response time to heating (Leigh et al. 2012). Thick leaves with high thermal mass exhibit slower response times to heating, thereby avoiding lethal temperatures, and reducing the temperature range unfavorable for photosynthesis (Leigh et al. 2012).

Specific leaf area serves as an indicator of leaf economic strategy associated with photosynthetic capacity. High SLA values indicate a larger surface area available for solar radiation absorption, which can result in increased leaf temperature (Michaletz et al. 2016). However, a higher SLA also allows for greater heat exchange with the atmosphere and increased transpiration (Wright et al. 2001).

Leaf dry matter content and SLA play fundamental mechanistic roles in thermal buffering and net carbon gain of leaves (Michaletz et al. 2016). Leaves with higher LDMC values (high dry mass) exhibit reduced heat transfer permeability but have a higher capacity to store heat energy. Conversely, leaves with low LDMC may heat up more rapidly in response to radiation, leading to greater thermal instability (Michaletz et al. 2015). Consequently, SLA and LDMC contribute to maintaining leaf temperature close to optimal levels for maximizing carbon assimilation (Michaletz et al. 2016).

Additionally, we measured leaf absorptance to shortwave radiation (*a*), which indicates how much radiant thermal energy the leaf can absorb. The higher the absorptance the higher the thermal energy absorbed and thus, the temperature (Lambers et al. 2019). Finally, stomatal conductance (g_s_) determines the transpiration capacity of leaves which in turn influences the cooling effect (Fauset et al. 2018).

To measure thermoregulatory morphological traits, we selected 10 mature and healthy individuals from each species within the hottest and the coldest part of the city (7 species × 10 individuals by species × 2 environments =140 trees). For each tree, we selected those branches wholly exposed to the sun and accessible from the ground, and we carefully collected five fresh, mature, healthy and fully expanded leaves exposed to direct sun. Leaves were stored in wet towels in Ziploc bags in a cooler to avoid dehydration while being transported to the laboratory of plant physiology of the Université du Quebec à Montreal to be processed within 24 h.

We measured LT (mm) using a digital micrometer (Mitutoyo, precision 0.0001 mm). To calculate LA (mm^2^) and LW (mm), we used the ImageJ software (http://rsbweb.nih.gov/ij/) and the LeafArea R package (Katabuchi 2015). Leaf width was calculated as the diameter of the largest circle capable of fitting within a leaf margin (Leigh et al. 2017). To calculate SLA (mm^2^ mg^−1^) and LDMC (mg g^−1^), we placed each leaf in a separate paper bag in the oven at 70 °C for 72 h before measuring dry weight using an analytical balance with a precision of 0.0001 g. Leaf absorptance to shortwave radiation (*a*) was calculated as *a =* 1 − leaf reflectance-leaf transmittance.

Leaf reflectance and leaf transmittance were measured with a CI-710 Miniature Leaf Spectrometer (Bioscience) over 400–1000 nm wavebands (Smith and Nobel 1977).

### In situ gas exchange and photosynthesis temperature responses in urban environments

Due to the limited duration of the summer and the time-consuming nature of the measurements, we selected a subset of five species from the seven species listed above to measure temperature responses of the light-saturated photosynthetic rate (A_net_): ACSA, CEOC, GLTR, QURU and TICO*.* We randomly selected five individual trees from each species in each environment (except for CEOC with three individuals). We selected low branches in each tree with leaves reachable from the ground, and one to three sun-exposed mature leaves from each tree to complete the temperature response curve following the general protocol of Slot and Winter (2017) (see Perez and Feeley 2020).

All leaves were measured in situ using a LICOR-6400 XT portable photosynthesis system (Licor, Lincoln, NE, USA). First, we measured leaf temperature with a digital infrared thermometer HT826 (CA, USA) to set the cuvette temperature at the same value, and then the leaf was allowed to acclimate to the chamber environment. All the measurements were done using the same parameters: the reference [CO_2_] was fixed at 400 p.p.m., the irradiance at the leaf surface at 1200 μmol photons m^−2^ s^−1^, the sample chamber’s relative humidity ≅ 50–60%, and a temperature treatment ranging from 20 to 40 °C, incremented by 2 °C intervals. Next, we visually inspected stabilization on these parameters to measure carbon assimilation rates (μ mol m^−2^ s^−1^) and stomatal conductance (g_s_) when the leaf was wholly acclimated to each target temperature. To increase the range of leaf temperature with the LICOR-6400 XT, we used a combination of ambient daytime temperatures variation between 09:00 and 15:00 h, and manipulation of block temperature of the Peltier-controlled leaf cuvette. We always measured photosynthesis on sunny days; in addition, while measuring photosynthesis, we also measured g_s_ and transpiration (E) in situ.

The temperature response of the light-saturated photosynthetic rate was fitted according to the model presented in June et al. (2004) and adapted by Slot and Winter (2017).


$$ P(T) = POpt\ast{e}^{-{\left(\frac{TLeaf- TOpt}{\varOmega}\right)}^{2}} $$

where P(T) is the net photosynthesis per unit LA, $POpt$ is the optimal carbon assimilation. T_Leaf_ is the leaf temperature. $\Omega$ describes the width of the curve’s peak and is the difference between TO_pt_ and the temperature at which PO_pt_ drops by about 37% of its value at TO_pt_.

### Photosynthetic heat tolerance (P_HT_)

Mature leaves were harvested for focal trees (same 10 individuals chosen for thermoregulatory morphological leaf traits) in the morning and were processed for heat tolerance between 10:00 and 16:00 h local time. We used F_V_/F_M_ values to estimate the P_HT_ following established protocols (Feeley et al. 2020, Perez and Feeley 2020). The temperature at which PSII performance begins to decrease is the critical temperature (T_crit_), the temperature at which ≥50% irrecoverable damage is T_50,_ and the temperature that leads to 95% irreversible and nearly complete heat damage to PSII is T_95_ (Perez et al. 2021).

To ensure the health of the samples, we measured the initial status of each individual leaf using the handheld fluorometer OS30P^+^ Opti-Science (Hudson, NH, USA). Five leaves from each individual tree were dark-adapted for 20 min. In the case of compound leaves, a random leaflet was chosen. After dark adaptation, we measured initial fluorescence emission (F_0_) and maximum total fluorescence (F_M_) to calculate the ratio of variable fluorescence (F_V_) as F_M_ − F_0_ to maximum leaf fluorescence as F_V_/F_M_. We ensured that F_V_/F_M_ was ~0.8 to continue the process. Subsequently, we cut leaf disks of ~2.0 cm in diameter using a hole punch, avoiding the central veins. For each temperature treatment, we placed three disks from each individual in Miracloth fabric to prevent anaerobiosis during the heat treatments (Perez and Feeley 2020); one layer of Miracloth covered the adaxial surface and three layers covered the abaxial disc surface. Each Miracloth packet was placed inside a waterproof Ziploc plastic bag (removing the air), and completely submerged for 15 min in preheated circulating water baths fixed at 22.0, 40.0, 42.2, 44.5, 47.0, 49.3, 51.5, 53.2 and 56.5 °C. Overall, we analyzed three disks by temperature per individual from both urban environments for a total of 3780 leaf disks. After the 15 min temperature treatment, we placed the disks into Petri dishes with moist towel paper to avoid dehydration. We allowed them to recover for precisely 24 h. Under low light and ambient temperature of ~24 °C. At the end of the recovery period, we dark-adapted the disks for 20 min to measure the final F_V_/F_M_.

To estimate P_HT_ for each species in UHI and the coldest part of the city, we modeled the relationship of F_V_/F_M_ vs treatment temperatures for each tree using a logistic non-linear least squares model with the ‘nls’ function in the *stats* R package (R Core Team 2021). We calculated T_50_ by predicting the temperature that caused damage in the 50% of PSII efficiency compared to the control temperature as:


$$ {\mathrm{T}}_{50} = \frac{\mathrm{\theta} 1\ }{1+\exp \left(-\left(\mathrm{\theta} 2+\mathrm{\theta} 3\ast \mathrm{Temperature}\right)\right)} $$

where θ_1_ is the control treatment (F_V_/F_M_ ≅ 0.8) and θ_2_ and θ_3_ are the intercept and slope from the logistic model (Perez and Feeley 2020). We generated bootstrapped means and 95% confidence level intervals of T_50_ by reiterating (with replacement) 100 times the nls model for each tree. Using the same model, we calculated T_crit_ and T_95_ as the temperature value where the F_V_/F_M_ begins to decline with 15 and 95% of PSII efficiency, respectively, compared with the control temperature.

### Leaf temperature modeling

We employed the leaf-scale energy balance model (Campbell and Norman 1998) to predict the temperature of a horizontal sun-exposed leaf and quantify how leaf-to-air temperature differences vary in response to urban environments. The model assumes that the leaf’s thermal radiation is balanced mainly by latent heat loss, sensible heat loss and emitted thermal radiation. Consequently, the leaf temperature can be effectively modeled by considering a combination of environmental factors and thermoregulatory traits.

To parameterize the leaf energy balance model, we used LW, g_s,_ and leaf absorptance (*a*). As environmental factors, we used the air temperature, relative humidity (%), and wind speed (ms^−1^) recorded using a digital anemometer Hyelec MS6252A (UK) when measuring the photosynthesis temperature response curves. Additionally, we used the solar irradiance which was collected with the HOBO data loggers located in the sampled trees in each urban environment. The model to calculate leaf temperature is described as:


$$ Tl= Ta+\frac{\gamma \ast }{s+\gamma \ast}\left[\frac{Rni}{gHR\ Cp}-\frac{VPD\ }{\rho \gamma \ast}\right] $$

where $Tl$ is the leaf temperature calculated with the model (°C), $Ta$ is air temperature (°C), $s$ is the slope of the saturation pressure curve, $Rni$ is the isothermal net radiation incident upon a leaf (W m^−2^) (i.e., the net radiation that the surface would receive if it had the same temperature as the air). $gHR$ is the sum of boundary layer conductance ($gHa)$ and radiative conductance ($gr)$ (mol m^−2^ s^−1^), $Cp$ is the heat capacity of dry air at constant pressure (29.3, Jmol^−1^C^−1^), VPD is vapor pressure deficit (KPa) and $\rho$ is the atmospheric pressure (101.3x${e}^{\left( altitude/8200\right)},\mathrm{KPa}$), $\gamma \ast$ is the modified psychrometric constant determined from the ratio of combined boundary layer and radiative conductance to stomatal conductance (Campbell and Norman 1998, Perez and Feeley 2020). For the calculation of the model, $\gamma \ast$, $Cp$, $\rho$ were constants. $\gamma \ast$ can be calculated by multiplying the psychrometric constant ($\gamma =$ 6.66 × 10^−4^ C^−1^) by the ratio of the radiative conductance ($gHR$) to the boundary layer conductance of water vapor (g_wv_). $gHR$= $gHa$+ $gr$*,* where $gHa$ is the boundary layer conductance for heat described as $gHa$= 1.4 × 0.135 $\sqrt[2]{u/d}$, *u* being the wind speed and *d* the effective LW in meters; and $gr$ is the radioactive conductance defined as $gr$ = $\frac{4\ \sigma\ Ta}{\ Cp}$, $\sigma$ being the Stefan–Boltzmann constant in W (5.67 × 10^−8^). The boundary layer conductance of water vapor (g_wv_) is defined as $\frac{0.5\ast \left(\frac{gs}{2}\right)\ast gva}{\left(\frac{gs}{2}\right)+ gva}$, where the boundary layer conductance for vapor is g_va_ = 1.4 × 0.147 $\sqrt[2]{u/d}$ (Campbell and Norman 1998, Perez and Feeley 2020).

### Data analysis

We ran a correlogram to detect autocorrelation in the time series of air temperature in each urban environment (Figure S1 available as Supplementary data at *Tree Physiology* Online). We then fitted ARIMA models separately to the two-time series (air temperature in the hottest and coldest city areas). Subsequently, we used a Wilcoxon test for non-normal distribution to compare the residuals between the two models to detect significative differences. We ran the analysis for the mean air temperature (T_air_Mean_ [°C]), daytime air temperature (T_air_dayMean_ [°C]), daytime maximum temperature (T_air_dayMax_ [°C]) and night temperatures (T_air_nightMean_ [°C]).

To assess our first question (QI: is there a response from of urban trees to the elevated temperatures within the UHI in their morphological or physiological traits?), we compared all the measured traits between urban environments. We conducted a nested analysis of variance (nested ANOVA) using mixed effect models through the lmer function from the “lme4” package in R (Bates et al. 2015). In the model, the urban environment (hottest [UHI] and coldest) was treated as a fixed effect, while species were considered a random effect. Size-related plant traits with power-law growth rate of the form Y = ax^b^ (LA, SLA, LT, LW) were log_10_ transform to normalize trait distribution. We used the ‘Anova’ function from the ‘car’ R package (Fox and Weisberg 2019) to compute the F-statistic and the degrees of freedom (DF) using the Kenward–Roger method. The *P*-value was calculated using the ‘lmerTest’ R package (Kuznetsova et al. 2017). For mean comparisons, we employed Tukey’s test using multcompt package in R (Hothorn et al. 2008). ANOVA assumptions were tested to run the model.

Next, we investigated whether the thermal adaptation responses to temperature variations between the urban environments are species-specific (QII). Because we employed a hierarchical sampling design, measuring five leaves from five individuals across seven species in two urban environments, we used a nested ANOVA, with the interaction of species and urban environment as fixed effect and individuals’ identity as random effect to account for intra-individual variation. The *P*-values and the DF were calculated using ‘lmerTest’ and ‘car’ packages, respectively. This analysis enables us to assess the effect of the urban temperature variation on thermoregulatory morphological traits by species and determine whether trees have developed passive thermoregulatory mechanisms through plastic changes in leaf traits.

To evaluate how much the tree species regulate leaf temperature and how leaf temperature varies from air temperature depending on the urban environment (QIII), we graphically compared the theoretical leaf temperature calculated with the energy balance model vs the observed leaf temperature. After, we ran a mixed analysis of covariance (mixed ANCOVA) using air temperature as covariable and individuals’ identity as random component to quantify the difference between the modeled leaf temperature within the hottest and coldest area using air temperature as a covariable. A higher decoupling of leaf temperature from air temperature could suggest difficulty in plant thermoregulation and acclimation.

To address QIV (do trees acclimate to higher temperatures within UHIs through physiological changes in their photosynthetic thermal optimum [T_opt_] and/or photosynthetic thermal tolerances [P_HT_]?) we used a two-way ANOVA to assess if tree species are showing physiological acclimation signals to higher temperatures within the UHI. In this case, first, we evaluated the effect of the urban environment and tree species on the variation in thermal tolerance (T_50_, T_crit_ and T_95_). Employing this analysis, we are able to assess the separated effect of each factor, and whether the effect of the urban environment on traits varies depending on the tree species. For post hoc comparisons between urban environments, we employed the emmeans test. To account for multiple comparisons, we applied the Bonferroni correction using the emmeans R package (Lenth et al. 2019). The emmeans test is particularly suitable for analyzing imbalanced data by adjusting the standard errors (SE) that arise due to variations in the number of observations. The normality and homoscedasticity assumptions were tested before conducting the analyses.

To analyze if there are signs of physiological acclimation in the photosynthetic thermal optimum (T_opt_), we used Welch’s t-test to evaluate the effect of urban environments on variation in T_opt_. This analysis calculates the degree of overlap of the SE for T_opt_ and optimal carbon assimilation (P_opt_) (Tarvainen et al. 2022). This test is appropriate for situations where the variances of the two groups are different, and the sample size between groups is unequal (case of CEOC). The fitted parameters of the model were significantly different when the test was higher than zero, as described in the following equation.


$$ \left|x1-x2\right|-2.99\times \sqrt{\left({SE}_1^2+{SE}_2^2\right) } > 0 $$

where $x1$ and $x2$ are the two parameters fitted with the model (e.g., P_opt,_ T_opt_) in each urban environment. The constant 2.99 is the t-score corresponding to *P* = 0.05 after adjustment to account for the multiple comparisons. ${SE}_1$ and ${SE}_2$ are the standard errors of the values’ fit (Tarvainen et al. 2022).

Furthermore, we analyzed the differences in both, stomatal conductance (g_s_) and transpiration (E) using a two-way ANOVA and Tukey’s test as post hoc method.

All the analyses were performed using the R version 4.0.0 (R Development Core Team 2021).

## Results

### Micro-urban climate

**Figure 2:**
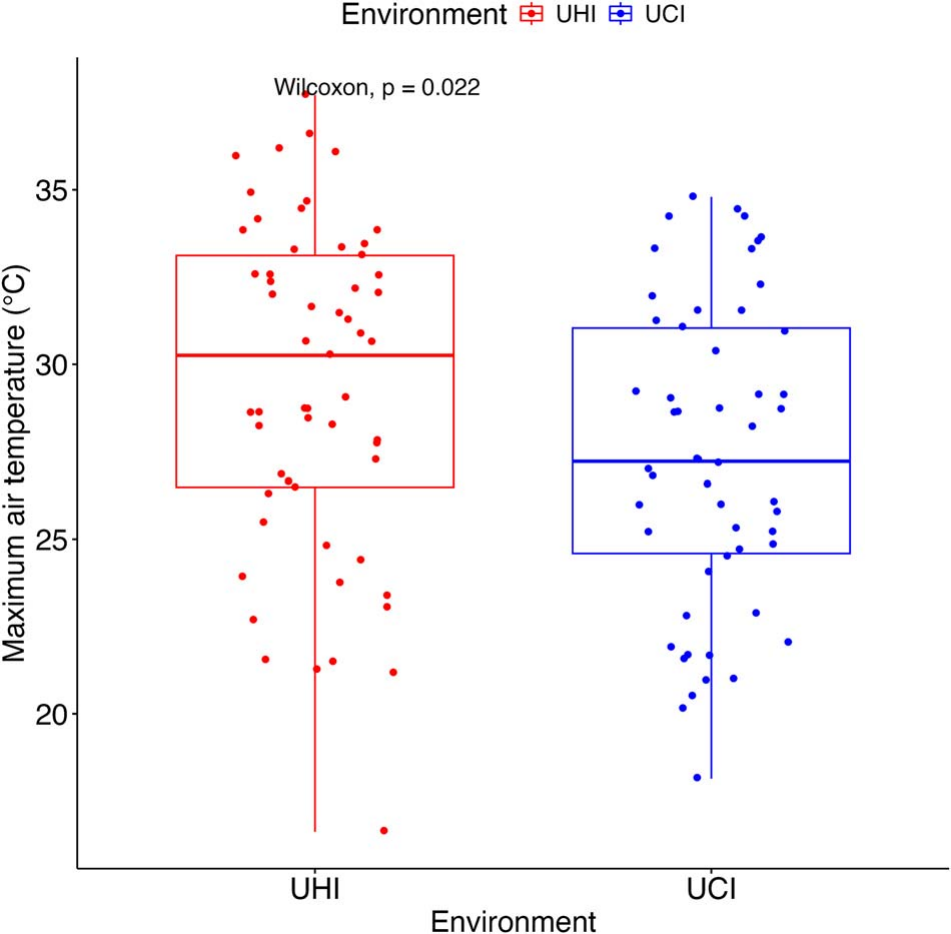
Maximum air temperature (°C) data within the hot vs cold sites in Montreal, Canada, from July to September 2021. Wilcoxon test is made with the residuals of the ARIMA models.

According to the Wilcoxon test on the residuals of the ARIMA models, all air temperature variables (T_air_Mean_, T_air_dayMean_, T_air_dayMax_, T_air_nightMean_) exhibited significantly larger values in the hotter part of the city, indicating the effect of temperature variation and the presence of the UHI (Figure S2, Table S1 available as Supplementary data at *Tree Physiology* Online). Particularly, the maximum air temperature, crucial for tree functioning, exhibited the greatest disparity between environments (Figure 2). With a warming of 3.4 °C in the UHI.

### Effects of heat on thermoregulatory traits

**Figure 3:**
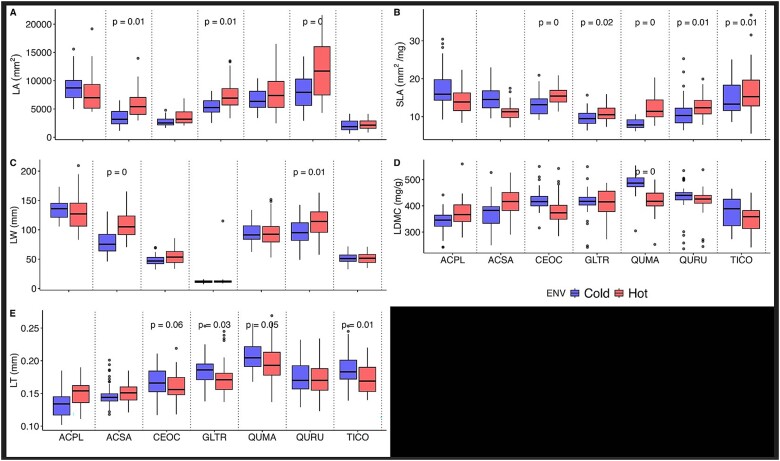
Variation in leaf morphological thermoregulatory traits between environments (hot vs cold) per specie in (A) LA, (B) SLA, (C) effective LW, (D) LDMC and (E) LT. Each box shows one species per environment, the line within each plot represents the median, the upper and lower limit of the boxes represent the 75th and 25th percentile, and the whiskers the 90th and 10th percentiles. Asterisks between boxes indicate significative differences resulting from the post hoc Tukey analysis between urban environments. The species are *A. platanoides* (ACPL)*, A. saccharinum* (ACSA)*, C. occidentalis* (CEOC)*, G. triacanthos* (GLTR)*, Q. macrocarpa* (QUMA)*, Q. rubra* (QURU) and *T. cordata* (TICO)*.*

The nested ANOVA analysis revealed that temperature significantly influences the LA, SLA and LW of the selected species (Table 1), showing that thermoregulatory leaf traits related with area were particularly sensitive to temperature variation between the UHI and coldest parts of the city. On the other hand, when we analyzed the effect of the temperature and the species on changes in thermoregulatory morphological traits using the nested ANOVA, our results indicated that the responses to elevated temperatures are highly species-specific (Figure 3). From all the traits, SLA had the highest intra-specific variation between the UHI and coldest areas, where five of the seven species showed significant increases within the UHI (Figure 3B). Regarding other traits, LT presented significant differences between the two environments in four of the seven species, with higher values within the UHI (Figure 3E). The LA showed increases within the UHI in three of the seven species (Figure 3A) but was the trait with the largest variation within the hottest part compared with the same species within the coldest part of the city (Table S2 available as Supplementary data at *Tree Physiology* Online). These results indicate that elevated temperatures highly affect morphological traits related to LA and mass. Finally, LDMC and LW were less affected by the temperature differences in the urban environment (Figure 3C and D).

**Table 1:** Analysis of variance table for the nested ANOVA of plant functional traits and urban environments (UHI and the coldest part of the city). In the ANOVA, urban environment (hot–cold) was included as fixed effect and species were included as a random effect. The DF were calculated using Kenward–Roger method. DF__random_ considers the structure of the random effects in the model. DF__fixed_ is 1 for all the models because the fixed effects are two treatments (hot–cold).

**Type of trait**	**Trait**	**Units**	**F-statistic**	***P*-value**	**DF_**_**random**_
**Morphologic**	**LA**	**mm** ^**2**^	**44.7**	**<0.001**	**643.06**
**Morphologic**	**LW**	**mm**	**21.5**	**<0.001**	**643.02**
**Morphologic**	**SLA**	**mm** ^**2**^ **mg**^**−1**^	**5.011**	**0.02**	**643.17**
Morphologic	LDMC	g g^−1^	3.20	0.07	643.41
**Morphologic**	**LT**	**mm**	**4.14**	**0.04**	**643.09**
**Photosynthetic**	**T**_**opt**_	**°C**	**3.55**	**0.06** ^**.**^	34.7
Photosynthetic	P_opt_	*μ*mol CO_2_ m^−2^ s^−1^	0.03	0.8	34.5
Thermal	T_crit_	°C	1.0	0.31	127.84
Thermal	T_50_	°C	0.47	0.40	127.15
Thermal	T_95_	°C	2.90	0.08	127.12

Bold values denote traits that were significant in the model.

### Modeled leaf temperature

**Figure 4:**
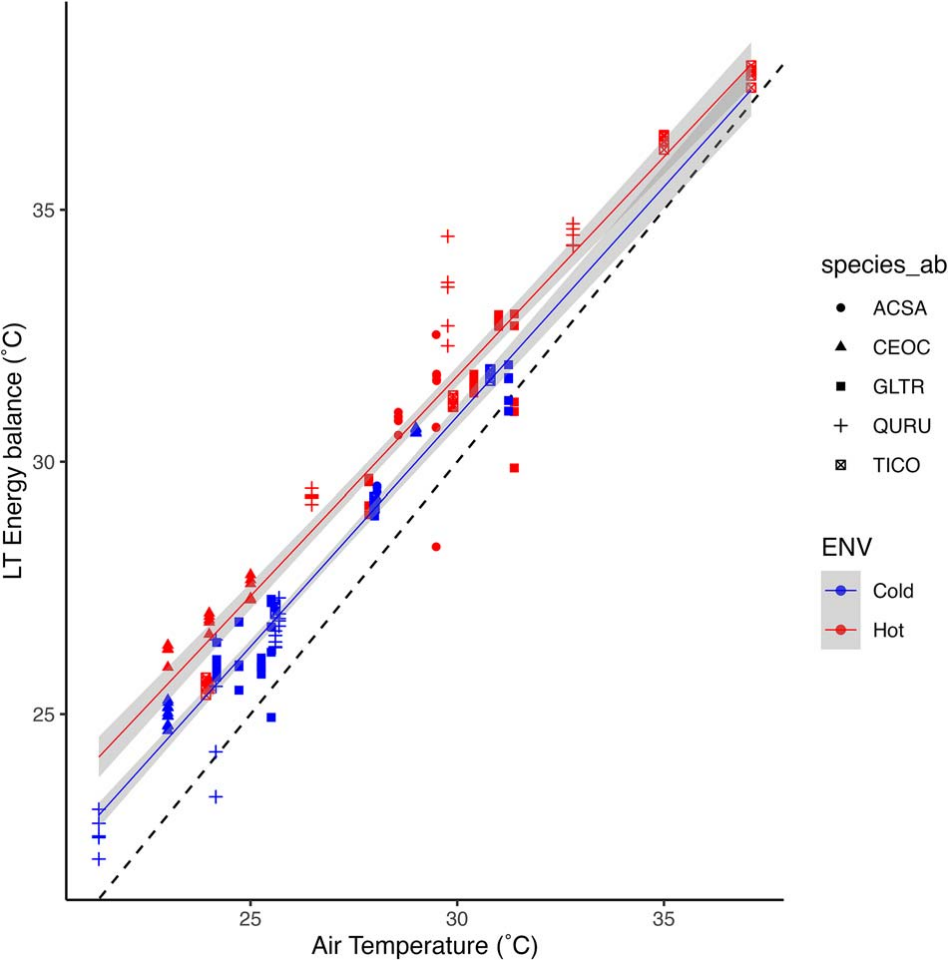
Energy balance estimations for leaf temperature (LT energy balance) vs air temperature for each individual and species within urban environments. The black dash is the y = x line. The mixed ANCOVA test to assess differences between the two slopes was *P* < 0.005.

**Figure 5:**
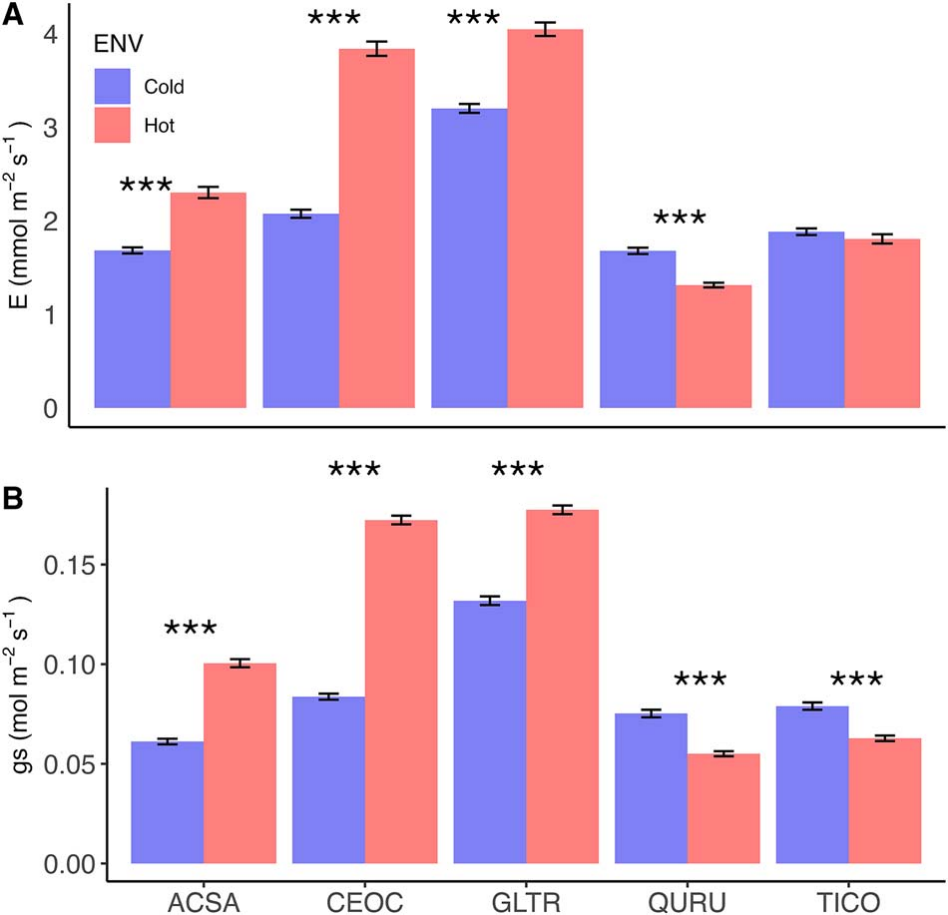
Between sites variation in (A) transpiration (E) and (B) stomatal conductance (g_s_). Data are shown as mean ± SE of the values. Asterisks indicate significant differences between urban sites within each species (*P* < 0.05). Statistical comparisons were made using Tukey’s honestly test. Abbreviation of species indicates *A. saccharinum* (ACSA), *C. occidentalis* (CEOC), *G. triacanthos* (GLTR), *Q. rubra* (QURU) and *T. cordata* (TICO).

We compared modeled leaf temperatures to air temperatures in both the UHI and the coldest parts of the city to evaluate individuals’ thermoregulation capabilities. Our analysis revealed that leaf temperatures for individuals in both the UHI and coldest part of the city consistently exceeded air temperatures (Figure 4). Notably, individuals located in the UHI exhibited even higher leaf temperatures than individuals in the coldest part of the city. The slopes of the relationship between leaf temperature differed significantly between the two urban environments (mixed ANCOVA *P*-value <0.005). Despite higher rates of transpiration and stomatal conductance observed within the UHI for nearly all species (Figure 5), leaf temperatures remained elevated compared to air temperatures. This indicates that heightened temperatures necessitate tree species to enhance evaporative cooling (E) and stomatal conductance (gs), yet leaf acclimation to these conditions appears limited.

### Photosynthetic heat tolerance (P_HT_)

**Figure 6:**
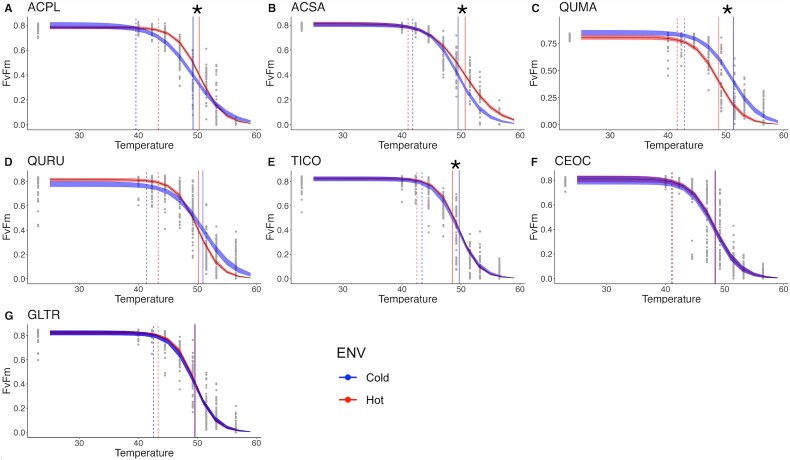
Non-linear least square models (nls) between F_V_/F_M_ and temperature to determine photosynthetic heat tolerance (P_HT_) for each species by the environment. Each dot represents individual leaf disks. The vertical continuous line represents T_50_ and dashes lines T_crit_. The 95% confidence intervals were calculated using 1000 bootstrapped temperature − _FV/FM_ nls models. The species are (A) *A. platanoides* (ACPL)*,* (B) *A. saccharinum* (ACSA)*,* (C) *Q. macrocarpa* (QUMA)*,* (D) *Q. rubra* (QURU)*,* (E) *T. cordata* (TICO), (F) *C. occidentalis* (CEOC)*,* (G) *G. triacanthos* (GLTR)*.* Asterisk denotes significative differences between urban environments in T_50_ from the two-way ANOVA.

Overall, we anticipated that all species would demonstrate increased P_HT_ as a typical response to elevated temperatures and heat acclimation. However, our nested ANOVA revealed no significant P_HT_ parameters (T_50_, T_crit_ and T_95_) as a general effect of temperature over all species together (Table 1). Upon examining the logistic models of F_v_/F_m_ in relation to temperature for each species between urban environments, we observed physiological acclimation in the T_50_ only in ACPL and ACSA to urban elevated temperatures. Conversely, QUMA and TICO displayed significantly lower heat tolerance in the hottest part of the city, while CEOC and GLTR exhibited nearly identical T_50_ in both environments (Figure 6, Figure S3a, Table S3 available as Supplementary data at *Tree Physiology* Online). Thus, only four of the seven species displayed significant differences between urban environments, with only two showing T_50_ acclimation. Furthermore, the T_50_ values for the seven urban tree species were ~50 °C, suggesting a likely upper limit by in P_HT_ temperature. The significant results at the species level, along with the lack of significance in the nested ANOVA conducted with the mixed model, suggest that certain species exhibit specific adaptations, whereas there may not be a general response across all species to elevated urban temperatures. This underscores the importance of species-level analyses in comprehending the impact of urban environments on tree thermal tolerance.

### Temperature responses of in situ gas exchange and photosynthetic thermal optimum (T_opt_)

**Figure 7:**
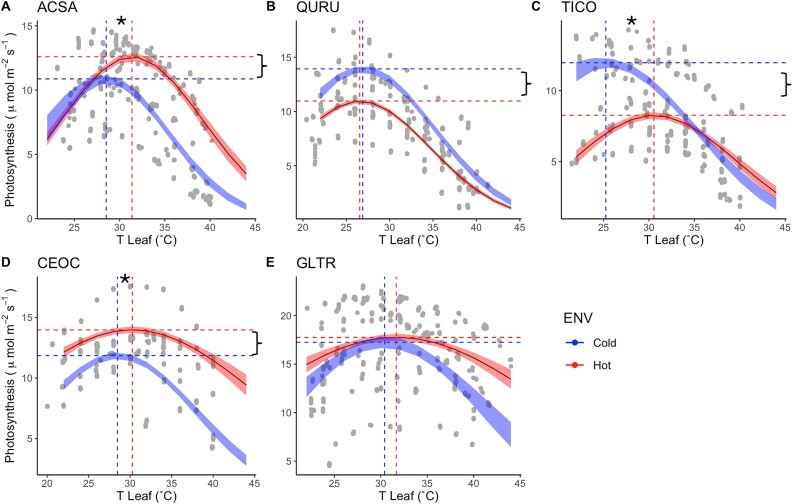
Light-saturated photosynthesis as a function of leaf temperature in the hottest part (UHI) and the coldest part of the city. (A) *Acer saccharinum* (ACSA), (B) *Q. rubra* (QURU), (C) *T. cordata* (TICO), (D) *C. occidentalis* (CEOC) and (E) *G. triacanthos* (GLTR). Discontinue vertical lines indicate the optimal temperature (T_opt_), and discontinue horizontal lines indicate optimal photosynthesis (P_opt_). Asterisks and brackets mean significant differences in T_opt_ and P_opt_, respectively, using the Welch’s t-test.

Although T_opt_ was not statistically significant in our nested ANOVA (*P*-value = 0.06), the marginal value suggests a trend of change among individuals located in different urban environments (Table 1, Table S3 available as Supplementary data at *Tree Physiology* Online). The Welch’s t-test, which analyzed intra-specific differences between urban environments, revealed significant differences among three of the five species selected to measure in situ photosynthetic responses to temperature between the UHI and coldest parts of the city (Figure 7, Figure S4a available as Supplementary data at *Tree Physiology* Online). Specifically, ACSA, CEOC and TICOR exhibited higher T_opt_ values in the UHI compared with the coldest part of the city, indicating photosynthetic thermal acclimation to elevated temperatures. On the other hand, P_opt_ was significantly higher in the UHI for ACSA and CEOC, but significantly lower for QURU and TICO (Figure 7, Figure S4b available as Supplementary data at *Tree Physiology* Online). The results, wherein species with higher T_opt_ values showed lower P_opt_ in the UHI, suggest that an increase in T_opt_ by acclimation does not necessarily lead to an increase in carbon assimilation.

Furthermore, significant differences were observed between urban environments for all species in both transpiration (E) and stomatal conductance (g_s_), except for E for TICO (Figure 5). ACSA, CEOC and GLTR exhibited higher E and g_s_ in the UHI, likely as a mechanism to facilitate cooling and sustain optimal carbon assimilation. Conversely, QURU and TICO displayed a slight decrease in both E and g_s_ within the UHI, likely, indicating the plants’ active response to mitigate dehydration (Figure 5A and B).

## Discussion

Urbanization significantly modifies the thermal environment for plants, impacting their physiological adaptation (Zipper et al. 2017). Greenhouse studies have documented plastic changes in leaf traits as a response to increased urban temperatures (Hara et al. 2021, Okubo et al. 2023). Yet, field studies involving mature trees are scarce. This study assessed plants’ thermoregulatory strategies and acclimation capacity in mature trees within the UHI and how affect their physiology. Overall, our results using the city of Montreal as scenario suggest that urban tree species have different ways of thermoregulating. One type of response we observed in our tree species to temperature increases was passive thermoregulation by adjustments in leaf morphology, mainly those related with area such as LA, SLA, but also those involving mass, such as LT. Another type of response was through an active thermoregulation by acclimating photosynthetic thermal optimum (T_opt_). Although we did not measure metabolism directly, it is important to note that changes in photosynthesis inevitably imply adjustments in metabolic processes (Yamori et al. 2014). Finally, photosynthetic heat tolerance (measured as T_crit,_ T_50,_ T_95_), did not show general trend of changes between UHI and the coldest part of the city. Although, this trait tends to be more conserved and retain its evolutionary climatic affinities (Bennett et al. 2021), it also has high adaptability and can show plastic changes under elevated temperatures (Zhu et al. 2020). We argue that, perhaps, this trait is not yet experiencing sufficient thermal stress to acclimatize across species. In fact, although two species showed increased T_50,_ other species showed a decreased T_50_ in the UHI. Our results also indicate the species-specific nature of thermal acclimation, underscoring the necessity for individualized species assessment in urban planning.

Previous studies show that slight increases in temperature, such as 0.78 to maximum 2.8 °C provoke significant modifications in physiological processes such as photosynthesis, as well as morphological traits (Zhu et al. 2020, Wei et al. 2023). In our study using actual urban microclimates, the maximum air temperature exhibited a difference of 3.4 °C between the UHI and the coldest parts of the city, which proved to be a critical factor influencing plant functioning. This slight temperature difference was enough to reveal significant physiological and morphological responses in urban trees, similar to those observed in previous studies, but evaluated in the specific context of urban environments.

### Changes in morphological leaf traits

Plants may respond to changes in temperature by adjusting their growth strategies and altering association among traits (Reich 2014). Because plant strategies are closely linked to resource acquisition and use, their variation is an important indicator of possible plant acclimation (Wang et al. 2022). Within the UHI, we found significant shifts for traits related to the area (LA, SLA, LW) and LT as a possible response to warmer urban temperatures, supporting our first hypothesis. However, the pattern of change in morphological leaf traits was contrary to our expectations in the second hypothesis. Instead of exhibiting shifts toward conservative strategies (lower values ​​of area-related traits), plants shifted toward acquisitive strategies within UHI (greater LA, SLA, LW and an increase in thickness).

In our case, larger values of LA, SLA and LW within the UHI may act as a mechanism to enhance the cooling effect through increased transpiration, thereby helping to mitigate leaf overheating (Fauset et al. 2018). The increases in traits related with LA are supported by increases in stomatal conductance and transpiration values for species with larger LAs within the UHI (e.g., *A. saccharinum, C. occidentalis, G. triacanthos*) (Figure 5). These coordinated increases in leaf traits likely result from an environment with rising temperatures but without water stress (Ibsen et al. 2023). Therefore, larger leaves can stay cooler and lose more heat through transpiration when there is sufficient water and adequate levels of solar radiation creating a unique plasticity pattern (Leigh et al. 2017, Ibsen et al. 2023). While municipal crews manage and water newly planted trees, this is not the case for mature trees, like those in our study. To account for potential differences in water availability, we specifically selected park trees, which are less likely to access supplemental water from nearby infrastructure. Thus, the rainfall in the city, particularly during the wettest months (May–September, totaling ~866 mm of precipitation), likely provides sufficient water to prevent drought stress in these trees (https://climate.weather.gc.ca/).

Leaf thickness, instead of increasing, decreased significantly for the same species with higher SLA within the UHI (Figure 3). Specific leaf area, a measure of the light-capturing area relative to biomass investment, tends to increase under elevated temperatures, allowing plants to enhance photosynthesis and cooling through transpiration (Xu et al. 2023). Furthermore, thinner leaves with higher SLA values are typically less costly to produce and can dissipate heat more effectively, especially when there is no water limitation (Ibsen et al. 2023). In urban areas with higher temperatures but sufficient water supply (such as through regular precipitation), plants might regulate heat by growing thinner leaves with increased SLA and optimize for light-capture, particularly in areas with taller buildings that create varying light dynamics. These plastic shifts in leaf traits are consistent with findings on urban trees, where trees increasing simultaneously carbon gain and the water transport capacity, indicating that urban trees can alter the water-use strategy meeting atmospheric demands (Rahman et al. 2020, Ibsen et al. 2023, Yin et al. 2024). While this study focuses on leaf morphological traits, it is important to recognize that other physiological and structural traits, such as stomatal density, cuticular conductance, turgor loss point and wood density, may also exhibit plasticity in response to temperature variation in urban environments. Further research into plastic responses of trees should investigate these traits along with a set of other environmental factors, such as humidity, wind speed, solar radiation and water access, to fully understand the mechanisms plants use to offset high temperatures in urban environments.

### Thermal tolerance (P_HT_) and photosynthetic thermal optimum (T_opt_)

The relationship between leaf temperature and photosynthesis is pivotal for understanding plant physiological acclimation within UHI and how elevated temperatures affect CO_2_ uptake in cities (Meineke et al. 2016, Percival 2023). Each of the urban tree species we analyzed had a unique photosynthetic acclimation response, highlighting the species-specific nature of physiological acclimations. *Acer saccharinum* and *C. occidentalis* showed a significant acclimation on T_opt_, also reflecting a higher photosynthetic rate at higher temperatures. This aligns with previous findings showing that urban flora can exhibit enhanced photosynthetic capacity at elevated temperatures due to acclimation (Hara et al. 2021). This particular response implies that these species might have an inherently high T_opt_, making them less sensitive to UHI effects. The photosynthetic acclimation of these species, coupled with higher stomatal conductance and transpiration, reflect higher metabolism in response to warming; such a response ensures better photosynthetic performance within UHI (Drake et al. 2018, Dusenge et al. 2019). However, it may also indicate that these species would not be able to maintain photosynthetic rate if high temperatures join with water deficit (Hara et al. 2021).

*Tilia cordata* exhibited a significantly higher optimal temperature for photosynthesis (T_opt_) within the UHI, but its net photosynthesis (A_net_) decreased substantially. This suggests either limited acclimation capacity (Kullberg and Feeley 2022) or that acclimation alone cannot fully offset the effects of rising temperatures, as it may not provide additional physiological benefits (Way and Yamori 2014). As a result, T_opt_ acclimation does not always improve A_net_ at elevated temperatures (Dusenge et al. 2019), and some species may even experience reduced growth in warmer environments (Meineke et al. 2016). A similar pattern was observed for *Q. rubra*; despite showing no acclimation in T_opt_, A_net_ was also significantly lower at elevated temperatures within the UHI. Both species demonstrated significantly reduced transpiration and stomatal conductance in the UHI, which can be attributed to their water-use strategy, being both categorized as isohydric species (Leuschner et al. 2019, Di Iorio et al. 2024). Species with this water-use strategy exercise strict control over their internal water status by closing stomata in response to high temperatures or limited water availability, which may result in photosynthesis being limited by CO_2_ supply. In these cases, rising temperatures can result in increased rates of photorespiratory CO_2_ release exceeding the carboxylation rates, causing decreases of A_net_ beyond an optimal temperature to which net photosynthesis is acclimated (Teskey et al. 2015).

Contrary to our hypothesis, we did not find that photosynthetic heat tolerance (P_HT_), particularly T_50_, acclimates to higher temperatures within the UHI as a general response for all the species, similar to previous studies developed in urban areas (Kullberg and Feeley 2022). Only *A. platanoides* and *A. sacharinnum* showed acclimation in their T_50_ within the UHI. On the other hand, the declining of T_50_ for *Q. macrocarpa* and *T. cordata* within the UHI suggests that these species may be more susceptible to heat stress. It has been proved that P_HT_ acclimates to seasonal temperature variation and to sustained changes in growth temperature, underlining that P_HT_ is highly temperature dependent (Zhu et al. 2020). To assess acclimation, it is crucial to evaluate the upper thermal limits of both leaf and air temperatures. In our study, we observed that the P_HT_ thresholds for nearly all species are close to ~50 °C, while summer air temperatures within the UHI rarely surpass 40 °C. Given this discrepancy, the prevailing air temperatures do not appear to reach the necessary levels to induce thermal stress to drive acclimation of P_HT_. As such, the relatively moderate urban warming observed in our case is insufficient to trigger or elucidate plant acclimation in this trait. Further studies in environments with more extreme urban heat or under experimental warming conditions may be necessary to understand the potential for thermal acclimation in these species fully.

### Leaf energy balance within UHI

Leaf temperature modeling revealed that the disparity between leaf and air temperature (ΔT) was more pronounced for individuals located within the UHI compared with those in the coldest part. This observation suggests that trees within the UHI may not be developing the capacity to acclimate to higher temperatures by adjusting their leaf energy balance. Our analysis of variation in morphological and physiological traits demonstrated changes in leaf morphology, such as increased LA and width within the UHI, as well as alterations in leaf physiology, including elevated transpiration cooling rates (E) and stomatal conductance (g_s_). These mechanisms play a crucial role in maintaining cellular homeostasis and metabolic function under heightened thermal conditions (Michaletz et al. 2015, 2016). However, the active augmentation of E and g_s_ does not fully offset the elevated temperatures to achieve complete acclimation to local conditions.

Leaf energy balance is governed by sensible and latent heat exchange, with the latter being regulated by plant available water, stomatal conductance and leaf-to-air VPD (Still et al. 2019). Consequently, in an urban setting such as ours, plants within the UHI will require greater water availability to withstand heat stress and potentially develop acclimation mechanisms. It is conceivable that if heat stress coincides with drought, stress levels in tree species will be exacerbated (Esperon-Rodriguez et al. 2021), and acclimation may not be feasible as drought reduces stomatal conductance and latent heat, thereby increasing leaf temperature. Urban water supply is often provided during establishment, when individuals are most susceptible to water constraints (Roman et al. 2014). Our findings underscore the importance of ensuring adequate water supply for mature trees as well, enhancing their acclimation capacity and cooling effect in warmer locales. This ensures that trees can continue to provide the ecosystem services upon which we rely now and in the foreseeable future.

## Conclusion

As cities continue to warm due to climate change, particularly within UHIs, understanding how urban trees acclimate to these novel conditions is crucial for urban planning (Teskey et al. 2015, Percival 2023). Our study on urban tree acclimation to elevated temperatures revealed highly species-specific responses, highlighting the complexity of plant adaptation in urban environments. We found that both LA and SLA increased, while transpiration rates rose, and LT decreased. Interestingly, only a subset of species showed acclimation in optimal photosynthetic temperature (T_opt_), associated with an increase in A_net_; meanwhile, some species, although acclimating the T_opt_, show decreases in A_net_. This highlights that photosynthetic acclimation to higher temperatures does not necessarily imply an increase in A_net_. Species with more conservative water-use strategies, such as isohydric species, tend to decrease carbon sequestration significantly due to stomatal control under urban environments with elevated temperatures. Overall, urban tree species exhibited no acclimation in photosynthetic heat tolerance (T_50_), likely due to the substantial thermal margin between their P_HT_ values and the ambient air temperatures, which remained ~10 °C lower. This significant buffer suggests that the air temperatures in urban environments do not impose sufficient thermal stress to trigger acclimation in this trait.

These findings together suggest that urban trees may prioritize strategies for leaf cooling, such as expanding LA and enhancing transpiration, rather than conserving water or thickening leaves in hotter, more humid climates. While our focus was on temperature, other factors like CO_2_ levels, irrigation and soil conditions could also influence these plastic responses. Future research should explore additional traits related to leaf acclimation—such as stomatal density, cuticular conductance and water-use efficiency—to develop a more comprehensive understanding of urban plant responses to UHI. Our study offers valuable insights, but we are aware that it was conducted over just 1 year. Long-term studies are encouraged to assess how plastic responses evolve across different environmental conditions, which would help forecast more accurate tree responses to urban stressors and UHI.

## Supplementary Material

Supplementary_information_Tree_physiology_tpae145

## Data Availability

Trait data that support our findings, including thermoregulatory morphological traits averaged to each individual tree, fluorescence and photosynthesis, are available in the figshare under the reserved DOI 10.6084/m9.figshare.25705995.
