# Supplementary material for: Changes in morphological and physiological traits of urban trees in response to elevated temperatures within an Urban Heat Island
Source: Tree Physiol. 2024 Nov 14;44(12):tpae145. doi: 10.1093/treephys/tpae145 (PMC11647596; doi:10.1093/treephys/tpae145)
Supplement: Supplementary_information_Tree_physiology_tpae145 [file supplementary_information_tree_physiology_tpae145.docx]

**Title:** Changes in morphological and physiological traits of urban trees in response to elevated temperatures within an Urban Heat Islands

**Running title:** Tolerance and responses of temperate trees to Urban Heat Islands

**Authors**: Johanna Andrea Martínez‐Villa^1*^, Alain Paquette^1^, Kenneth J. Feeley^2^, Paula Andrea Morales-Morales^3^, Christian Messier^1^, Sandra M. Durán^4^.

^1^Département des sciences biologiques, Centre for Forest Research, Université du Québec à Montréal, Montréal, Québec, Canada.

^2^Biology Department, University of Miami, Coral Gables, FL 33146, USA.

^3^Departamento de Ciencias Forestales, Universidad Nacional de Colombia- Sede Medellín, Colombia

^4^Department of Forest and Rangeland Stewardship, Fort Collins, Colorado, 80523 USA.

**SUPPLEMENTARY INFORMATION**

**TABLES**

**Table S1.** Air temperature (T_air_Mean_, °C), daytime air temperature (T_air_dayMean_, °C), maximum air temperature (T_air_dayMax_, °C), and air temperature at night (T_air_nightMean_, °C). Each environment has five HOBBO sensors recording temperature from July to the end of September 2021. The day temperature was calculated from 9 to 17 h. The night temperature was calculated from 17 to 23 h. Values in brackets are standard deviations. In parentheses are the p-values from the Wilcoxon test. Hottest and Coldest denotate the UHI and the coldest part of the city.

| **Urban**  **Environment** | **T_air_Mean_**  (*P-value<0.001*) | **T_air_dayMean_**  (*P-value<0.001*) | **T_air_dayMax_**  (*P-value*=0.02) | **T_air_nightMean_**  (*P-value*<0.001) |
| --- | --- | --- | --- | --- |
| Hottest | 23.3 [±4.3] | 25.9[±4.4] | 37.8 [±2.3] | 23.0 [±3.8] |
| Coldest | 22.0[±4.6] | 24[±4] | 34.4 [±2.1] | 22.1 [±3] |

**Table S2.** Thermoregulatory traits by species in two urban environments, The hottest (UHI) and the coldest part of the city (UCI). Means ± SD are represented. Species are: *Acer platanoides* (ACPL)*, Acer saccharinum* (ACSA)*, Celtis occidentalis* (CEOC)*, Gleditsia triacanthos* (GLTR)*, Quercus macrocarpa* (QUMA)*, Quercus rubra* (QURU) and *Tilia cordata* (TICO)*.*

| **Species** | **ENV** | **L. Abs** | **LA (mm^2^)** | **SLA** | **LW (mm)** | **LDMC (mg g^-1^)** | **LT(mm)** |
| --- | --- | --- | --- | --- | --- | --- | --- |
| **ACPL** | **UCI** | 0.295 +0.02 | 8833 + 1439 | 17.5+4.9 | 135+ 7.17 | 236+62.9 | 0.13+0.01 |
|  | **UHI** | 0.396 0.01 | 7682+ 1593 | 14.2+3 | 128 + 17 | 369+57.4 | 0.15+0.01 |
| **ACSA** | **UCI** | 0.322 +0.02 | 3570 + 1053 | 14.5+2 | 79.5 + 15.8 | 375+42.3 | 0.14+0.01 |
|  | **UHI** | 0.322 0.01 | 5669 + 1893 | 11.1+1.9 | 106 + 18.2 | 423+45.1 | 0.15+0.01 |
| **QUMA** | **UCI** | 0.318+0.02 | 6695.6+1139 | 8.08+1.1 | 94.3+10.6 | 504.5+22.2 | 0.20+0.01 |
|  | **UHI** | 0.308+0.01 | 7946+3072 | 12.3+2.4 | 97.0+15.8 | 416.7+66 | 0.19+0.03 |
| **QURU** | **UCI** | 0.281+0.01 | 8273.8+2924 | 11.0+3.1 | 96.1+16.9 | 434.5+43.6 | 0.17+0.02 |
|  | **UHI** | 0.271+0.05 | 11960+4506 | 12.6+2.5 | 111.54+22.6 | 431.1+39.4 | 0.17+0.02 |
| **TICO** | **UCI** | 0.314+0.01 | 2084.8+853 | 14.6+3.9 | 51.7+8.26 | 381.3+56.9 | 0.18+0.02 |
|  | **UHI** | 0.274+0.05 | 2332.2+789 | 16.6+5.6 | 51.8+7.6 | 358.2+57.5 | 0.17+0.02 |
| **CEOC** | **UCI** | 0.302+0.01 | 2742.4 +567 | 12.8+2 | 48.3+6.3 | 420.3+27 | 0.17 +0.02 |
|  | **UHI** | 0.297+0.01 | 3629.3+1081 | 14.5+2.1 | 54.9+10.4 | 387.3+31 | 0.16 +0.01 |
| **GLTR** | **UCI** | 0.327+0.02 | 5336.6+901 | 9.7+1.5 | 11.7+1.2 | 418.7+33.8 | 0.18+0.01 |
|  | **UHI** | 0.303+0.05 | 7477.9+1959 | 10.9+2 | 14.0+6.6 | 419.9+37.1 | 0.17+0.03 |

**Table S3.** Parameters (±SE) estimated for thermal tolerance and net photosynthesis in response to temperature for seven urban species in hottest and coldest part of the city. Species are: *Acer platanoides* (ACPL)*, Acer saccharinum* (ACSA)*, Celtis occidentalis* (CEOC)*, Gleditsia triacanthos* (GLTR)*, Quercus macrocarpa* (QUMA)*, Quercus rubra* (QURU) and *Tilia cordata* (TICO)*.*

| **Species** | **F_v_/F_m_** | | | | | | **Photosynthesis** | | | | | | | | | | |
| --- | --- | --- | --- | --- | --- | --- | --- | --- | --- | --- | --- | --- | --- | --- | --- | --- | --- |
|  | **UHI** | | | **UCI** | | | | | **UHI** | | | **UCI** | | | |  |  |
|  | **T_crit_** | **T_50_** | **T_95_** | **T_crit_** | **T_50_** | **T_95_** | | **T_opt_** | | **P_opt_** | **T_opt_** | | **P**_opt_ |  |  |  |  |
| **ACPL** | 43.3±0.8 | 50.4±0.1 | 56.3±0.7 | 39.5±0.7 | 49.4±0.4 | 57.7±1.1 | | - | | - | - | | - |  |  |  |  |
| **ACSA** | 41.1±0.7 | 50.7±0.2 | 58.9±0.3 | 41.7±0.6 | 49.5±0.3 | 56.1±0.4 | | 31.36±0.1 | | 12.59±0.1 | 28.52±0.4 | | 10.87±0.1 |  |  |  |  |
| **QUMA** | 41.6±0.7 | 48.8±0.1 | 54.8±0.5 | 42.9±0.6 | 51.1±0.4 | 58.0±0.9 | | - | | - | - | | - |  |  |  |  |
| **QURU** | 43.3±0.6 | 50.2±0.2 | 55.7±0.7 | 41.3±1.2 | 51.0±0.3 | 59.2±0.6 | | 26.55±0.1 | | 10.97±0.1 | 26.90±0.2 | | 13.93±0.2 |  |  |  |  |
| **TICO** | 42.6±0.6 | 48.5±0.2 | 53.5±0.4 | 43.4±0.5 | 49.7±0.1 | 54.9±0.5 | | 30.54±0.2 | | 8.27±0.1 | 25.25±0.6 | | 11.97±0.2 |  |  |  |  |
| **CEOC** | 41.2±0.7 | 48.5±0.5 | 54.6±0.6 | 41.0±0.7 | 48.4±0.7 | 54.6±0.5 | | 30.27±0.3 | | 13.97±0.1 | 28.47±0.1 | | 11.85±0.1 |  |  |  |  |
| **GLTR** | 43.3±0.5 | 49.7±0.1 | 54.8±0.3 | 42.5±0.3 | 49.3±0.3 | 55.1±0.5 | | 31.66±0.4 | | 17.75±0.1 | 30.41±0.3 | | 17.25±0.3 |  |  |  |  |

**FIGURES**

**
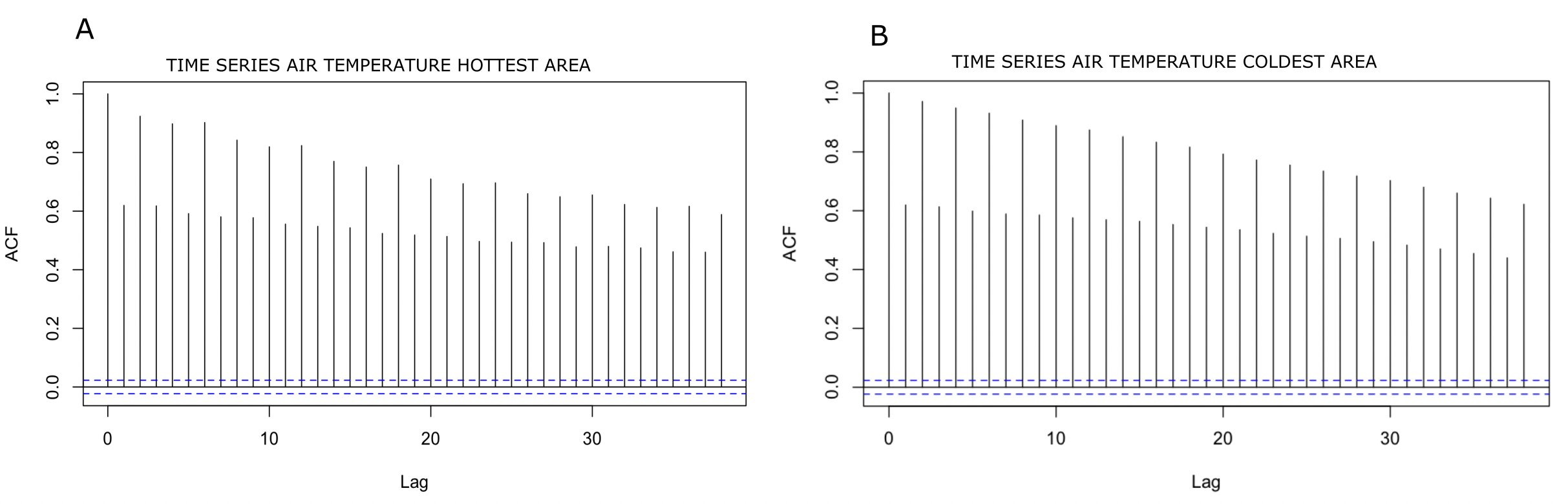
**

**Figure S1)** Correlogram made with the air temperature time series within the A) hottest part (Urban Heat Island [UHI]) and B) the coldest part of the city. The x-axis represents the lag, and the y-axis represents the autocorrelation. The height of the peaks indicates the strength of the autocorrelation. Blue lines denotates the confidence intervals.


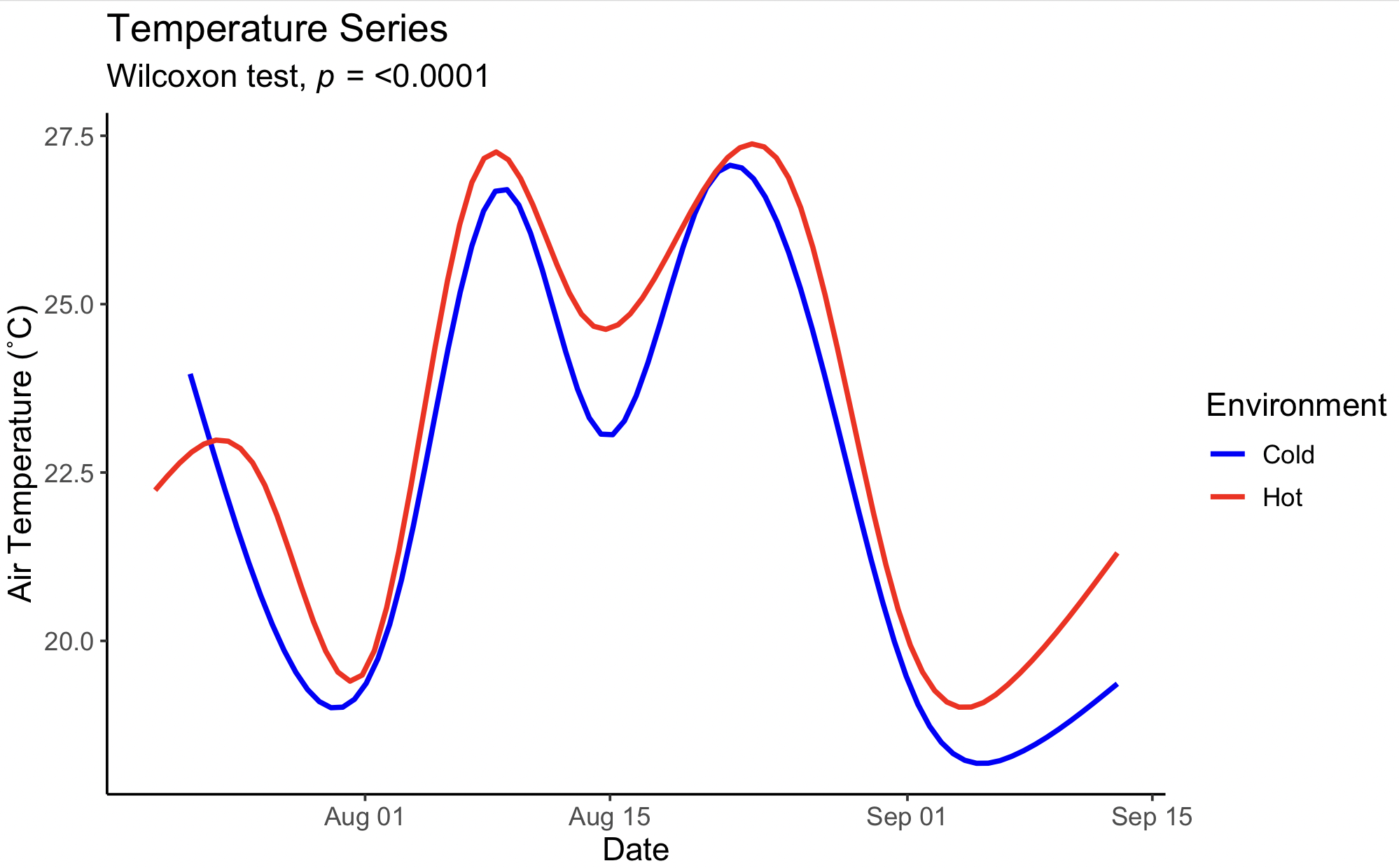


**Figure S2)** Time series variation in air temperature (ºC) (including day and night) from the HOBBO stations within the hottest (red) and coldest (blue) part of the city during the field camping (from July to September 2021). P<0.05 denotes the result from the Wilcoxon-test between urban environments.

**
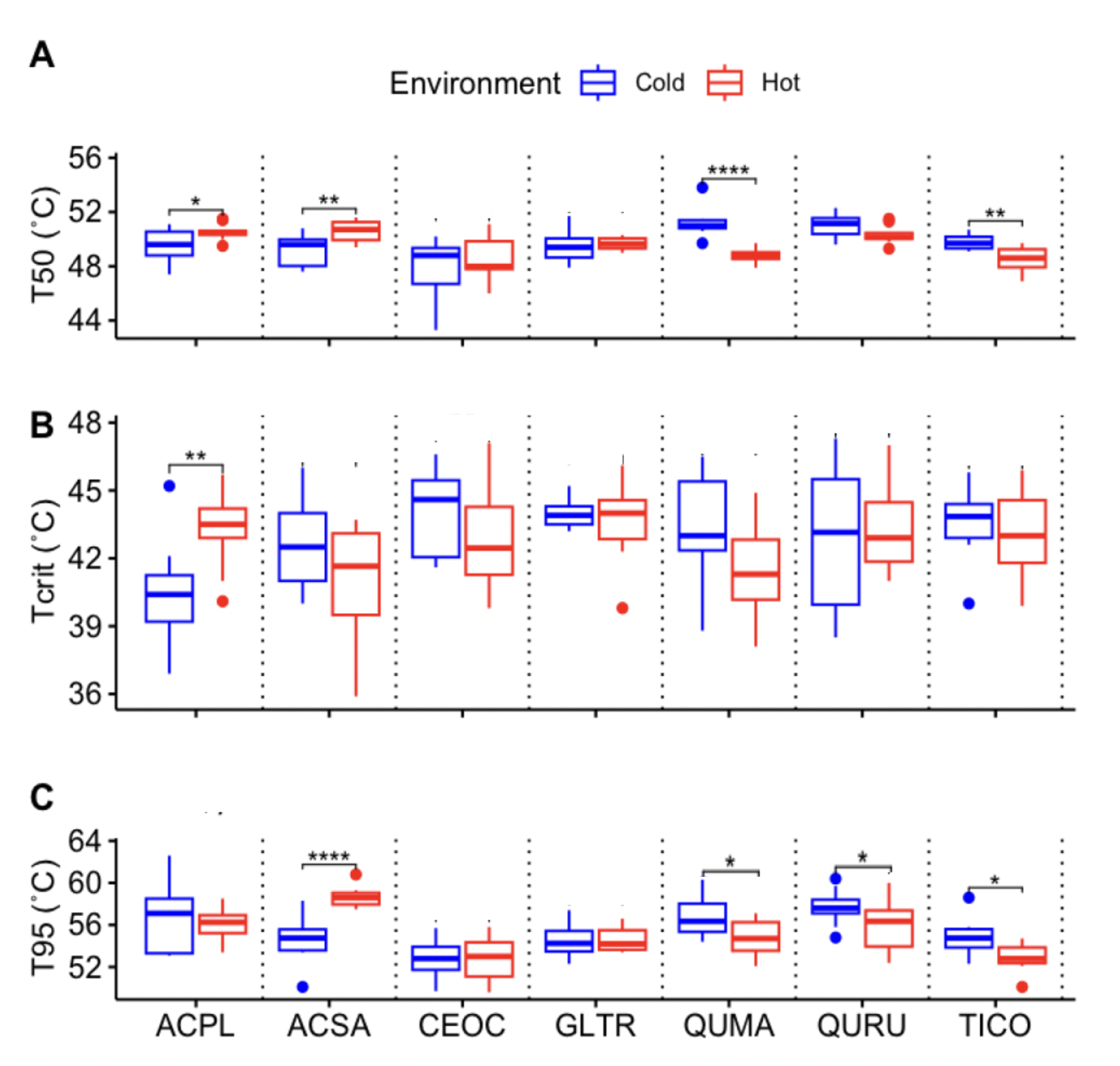
**

**Figure S3)** Two-way analysis of variance for the photosynthetic thermal tolerance parameters A) T_50_, B) T_crit,_ and C) T_95_ of seven species in two urban environments. Each panel shows one specie in the two environments and each box shows the median value, the upper and lower limit of the boxes represent the 75^th^ and 25^th^ percentile, and the whiskers the 90 ^th^ and 10 ^th^ percentiles. Red color denotes the hottest part of the city (Urban Heat Island [UHI]) and blue, coldest part. Asterisks between boxes indicate significative differences resulting from the Tukey *post-hoc* analysis between urban environments. The species are *Acer platanoides* (ACPL)*, Acer saccharinum* (ACSA)*, Celtis occidentalis* (CEOC)*, Gleditsia triacanthos* (GLTR)*, Quercus macrocarpa* (QUMA)*, Quercus rubra* (QURU) and *Tilia cordata* (TICO)*.*


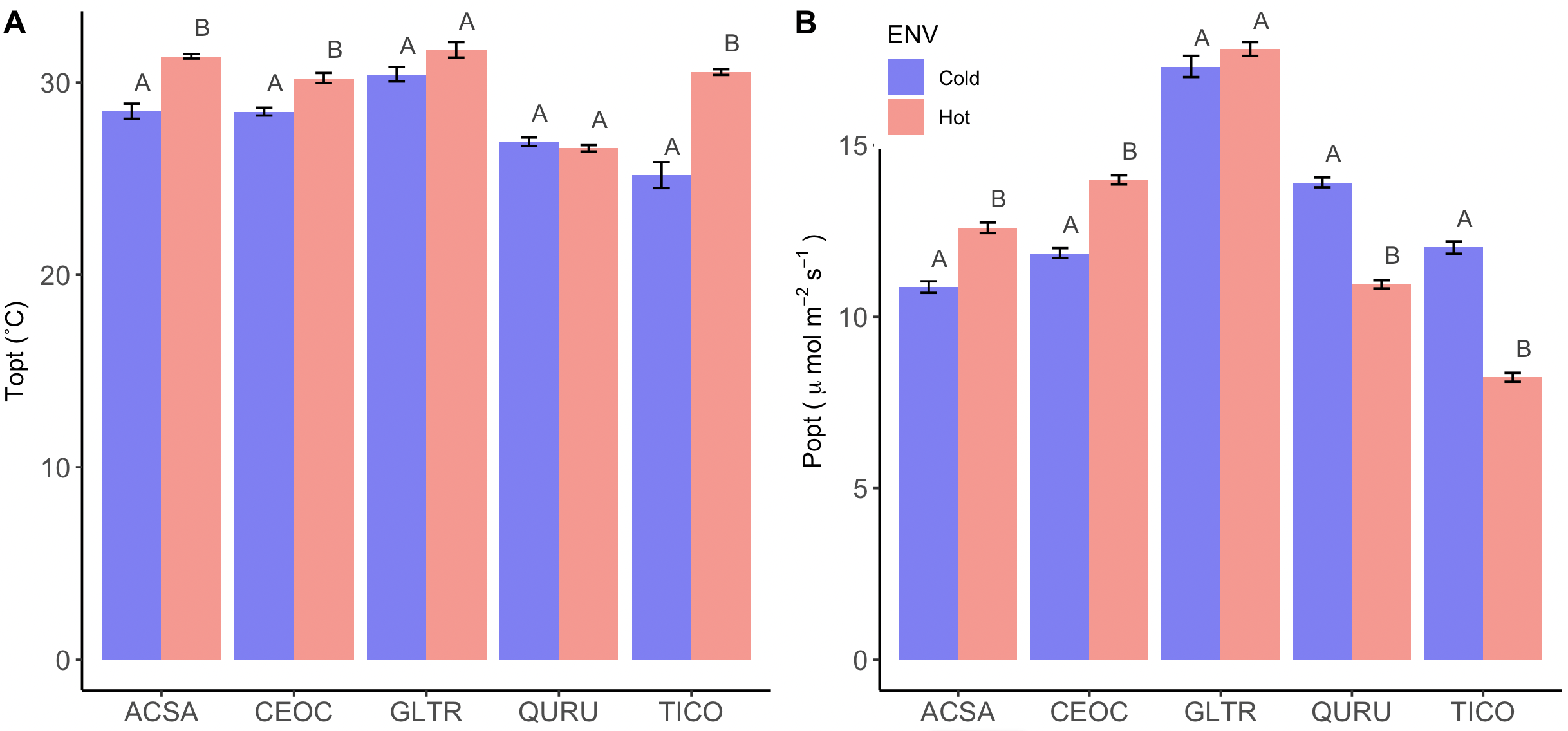


**Figure S4)** Between sites variation in A) T_opt_ and B) P_opt._ Data are shown as mean ± SE of the fitted temperature and assimilation values. The upper-case letter indicates significant differences urban between sites within each species (P<0.05). Welch’s t-test was used for statistical comparison among groups. Abbreviation of species indicates *Acer saccharinum (*ACSA), *Celtis occidentalis* (CEOC), *Gleditsia triacanthos* (GLTR), *Quercus rubra* (QURU) and *Tilia cordata* (TICO).
